# Tunable atomic spin-orbit coupling synthesized with a modulating gradient magnetic field

**DOI:** 10.1038/srep18983

**Published:** 2016-01-11

**Authors:** Xinyu Luo, Lingna Wu, Jiyao Chen, Qing Guan, Kuiyi Gao, Zhi-Fang Xu, L. You, Ruquan Wang

**Affiliations:** 1 State Key Laboratory of Low Dimensional Quantum Physics, Department of Physics, Tsinghua University, Beijing 100084, China; 2 Beijing National Laboratory for Condensed Matter Physics, Institute of Physics, Chinese Academy of Sciences, Beijing 100080, China; 3 MOE Key Laboratory of Fundamental Physical Quantities Measurements, School of Physics, Huazhong University of Science and Technology, Wuhan 430074, China; 4 Collaborative Innovation Center of Quantum Matter, Beijing, China

## Abstract

We report the observation of synthesized spin-orbit coupling (SOC) for ultracold spin-1 ^87^Rb atoms. Different from earlier experiments where a one dimensional (1D) atomic SOC of pseudo-spin-1/2 is synthesized with Raman laser fields, the scheme we demonstrate employs a gradient magnetic field (GMF) and ground-state atoms, thus is immune to atomic spontaneous emission. The strength of SOC we realize can be tuned by changing the modulation amplitude of the GMF, and the effect of the SOC is confirmed through the studies of: 1) the collective dipole oscillation of an atomic condensate in a harmonic trap after the synthesized SOC is abruptly turned on; and 2) the minimum energy state at a finite adiabatically adjusted momentum when SOC strength is slowly ramped up. The condensate coherence is found to remain very good after driven by modulating GMFs. Our scheme presents an alternative means for studying interacting many-body systems with synthesized SOC.

Spin-orbit coupling (SOC), as is often referred to in condensed matter physics, couples the spin of a particle to its orbital degrees of freedom. It is believed that SOC constitutes an important ingredient for quantum simulation with ultracold atoms^1^,^2^,^3^,^4^. Research on SOC is an active area due to its ubiquitous appearance in condensed matter phenomena, such as topological insulator^5^,^6^, spin Hall effect^7^,^8^, and spintronics^9^. In contrast to solid-state materials, where SOC originates from the orbital motion of electrons inside a crystal’s intrinsic electric field, the coupling between atomic spin and its center of mass motion has to be engineered artificially. In recent years we have witnessed great successes of artificial atomic gauge fields^10^,^11^,^12^,^13^,^14^,^15^,^16^,^17^,^18^,^19^. A popular scheme employs Raman laser fields^11^ to couple two atomic ground states forming a pseudo-spin-1/2 system, leading to a SOC with equal Rashba^20^ and Dresselhaus^21^ contributions. This is routinely used nowadays for both bosonic^11^,^14^ and fermionic^15^,^16^ alkali atom species. More general forms of SOC are pursued actively in a variety of settings^22^,^23^,^24^,^25^, which together with the above well understood Raman scheme^26^,^27^,^28^,^29^,^30^,^31^ significantly expand the scopes of quantum simulation using synthetic gauge fields in ultracold atoms, fostering exciting opportunities for observing novel quantum phenomena with ultracold atoms^32^,^33^,^34^,^35^,^36^,^37^,^38^,^39^,^40^,^41^,^42^.

The Raman scheme, pioneered by the Spielman group^11^, makes use of coherent atom-light interaction. As pointed out by several authors^2^,^4^,^43^, spontaneous emissions, nevertheless, come into play in the presence of even far off-resonant laser fields, which give rise to heating or atom loss. The effective spontaneous emission rate (heating rate) and the effective Rabi frequency for the Raman spin flip process both scale as the ratio of laser power to detuning squared, and are respectively proportional to the excited state spontaneous emission rate and fine structure splitting. Thus spontaneous emission cannot be suppressed with increased detuning. At a fixed effective Rabi frequency, heating will be stronger for K atoms than for Rb and Cs atoms, because the excited state fine structure splitting for K atom is smaller. The situations are worse for Na and Li atoms, whose even smaller fine structure splittings essentially rule out the possibility for observing many body phenomena with SOC from the Raman scheme. Alternative ideas less affected by atomic spontaneous emissions are proposed, such as using narrow-line transitions in high spin atoms^44^,^45^ or manipulating a spin-dependent tunneling without spin-flip in an optical lattice tilted by a static gradient magnetic field (GMF)^43^. Besides heating from atomic spontaneous emission, the SOC strength realized in the Raman scheme is difficult to be tuned continuously in an experiment because it is determined by the photon recoil momentum and the intersection angle between the two Raman laser beams. An idea based on periodically modulating the effective Rabi frequency can reduce the synthesized SOC strength^46^, which is successfully realized in recent experiments^47^.

To overcome the restrictions due to atomic spontaneous emission and to enhance tunability, one can seek out spin-dependent interactions between atoms and magnetic fields to synthesize SOC, as was proposed by Xu *et al.*^48^ using repeated GMF pulses and by Anderson *et al.*^49^ using modulating GMFs. A GMF provides a spin-dependent force, which over times leads to a spin (atomic internal state) dependent momentum (spatial/orbital degrees of freedom) impulse, hence gives rise to SOC. An analogous protocol is also proposed to generate SOC for atoms in an optical lattice^50^. These ideas can be applied to all spinful atoms^48^,^49^. A modulating GMF is recently used to produce a state dependent optical lattice for ultracold fermionic atoms^51^. The procedure for obtaining an effective Hamiltonian from such a periodically driven quantum system is developed in ref. 52. A non-uniform magnetic field is also widely used to couple electronic spin and orbital degrees of freedom in the condensed matter systems^53^,^54^,^55^.

This article reports our observation of a tunable SOC with equal Rashba and Dresselhaus contributions in a spin-1 ^87^Rb atomic Bose-Einstein condensate (BEC) synthesized by modulating a one dimensional (1D) GMF^48^,^49^. In the absence of other non-commuting interaction terms, The SOC with strengh *k*_so_ we report corresponds to a spin-dependent momentum shift ∝ *m*_*F*_*k*_so_ to the single-particle dispersion curves for spin component *m*_*F*_, which gives rise to the three crossing dispersion curves for the three spin components of spin-1 atoms. The synthesized SOC is confirmed through the following two observations: first, we measure the excitation of the collective dipole mode of a condensate in a harmonic trap after abruptly turning on the SOC; and second, we achieve the adiabatic loading of condensed atoms into the minimum energy state at shifted momentum when SOC is gradually turned on by slowly ramping up the amplitude of the modulating GMF.

## Results

**Figure 1:**
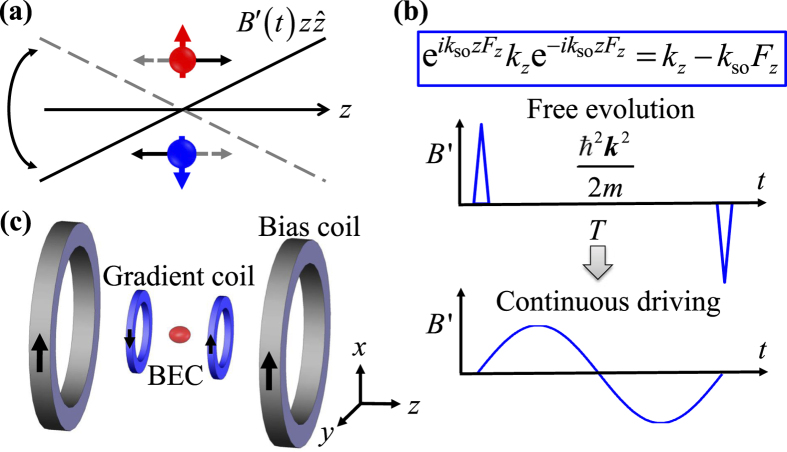
A schematic illustration of SOC synthesized from a periodic GMF. (**a**) A periodically modulated GMF with zero average 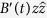 imparts opposite forces (black arrows or gray dashed arrows at different times) to the 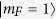 (red disk and arrow) and 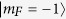 (blue disk and arrow) states of the *F* = 1 hyperfine manifold. (**b**) Each modulation period is composed of a pair of short opposite GMF pulses (blue triangles), which provide impulses ±
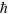
*k*_so_ with free evolution sandwiched in between, translating canonical momentum *k*_*z*_ → *k*_*z*_ − *k*_so_*F*_*z*_, leading to the SOC as shown in the blue rectangular box^48^. The continuous driving limit of a sinusoidal modulation with zero average is adopted in our experiment for its better technical stability^49^. (**c**) The experimental setup involves bias (gray) and gradient (blue) magnetic coils. A BEC (red football) is placed at the center of the gradient coils and aligned along the bias field.

Our experiments synthesize an effective 1D SOC by applying a time-periodically modulating 1D GMF 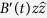 with zero average^48^,^49^, as illustrated in Fig. 1. The GMF provides a spin-dependent force 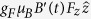 for a spin *F* atom (with mass *m*). Here, *μ*_*B*_ is the Bohr magneton, *g*_*F*_ is the Lande g-factor and *F*_*x*,*y*,*z*_ denotes the *x*-, *y*-, and *z*-component of spin vector **F**. Although the net impulse over one period is zero, the accumulated distance an atom moves depends on its spin state, which implies the atom acquires a spin dependent group velocity. Thus atomic spin and its center-of-mass motion (orbital) is coupled by GMF pulses. The origin for the synthesized 1D SOC can be understood more straightforwardly when we consider the extreme case where each period contains a pair of two opposite GMF pulses of impulse ±
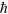
*k*_so_ capping the ends of free evolution over time *T*. These two pulses enact a unitary transformation, which displaces the canonical momentum by a spin-dependent quantity, *i.e.*, 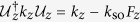 where 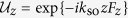, as shown in Fig. 1(b). Hence, the effective Hamiltonian 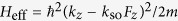 results from a spin dependent momentum shift to the free particle Hamiltonian 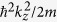. For the sinusoidal modulating GMF 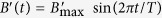 used in our experiments, the atomic dynamics is governed by 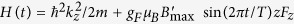. Based on the Floquet theory, the effective time-independent Hamiltonian *H*_eff_ is given by (more details in supplementary material)


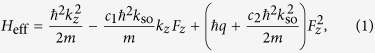


where 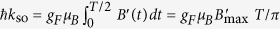, *c*_1_ = 1/2, and *c*_2_ = 3/8. 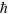
*q* is the quadratic Zeeman shift (QZS) of the bias field used for selecting the 1D GMF from a 3D quadrupole magnetic field (see supplementary material). The *c*_1_ and *c*_2_ terms on the rhs of Eq. (1) describe the synthesized SOC and the effective QZS, respectively. The latter together with 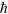
*q* can be further tuned to zero or negative by an off-resonant dressing microwave field^56^. The above physical picture remains approximately valid in the presence of an external harmonic trap if the modulation frequency *ω*_mod_ = 2*π*/*T* is far greater than the trap frequency.

Our experiments are performed in a single chamber BEC setup as described elsewhere^57^. We create a ^87^Rb BEC of 1.2 × 10^5^ atoms in state 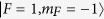 in a crossed dipole trap with trapping frequencies 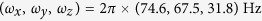, as illustrated in Fig. 1(c). The 1D GMF 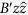 is implemented by a combination of a 3D quadrupole magnetic field 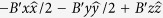 and a 5.7 Gauss bias field 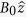, whose linear and quadratic Zeeman shifts are (2*π*) 4 MHz and (2*π*) 2.34 kHz, respectively (see supplementary material). The modulation frequency *ω*_mod_ for the GMF is (2*π*) 1.0 kHz unless stated otherwise. To generate a SOC strength *k*_so_ = 1 *k*_*L*_ = 8.0554 *μm*^−1^, where 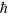
*k*_*L*_ = 2*π*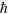/*λ*_*L*_ is the one photon recoil momentum at a laser wavelength *λ*_*L*_ = 780 nm, the required peak magnetic field gradient 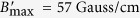. The centers of the gradient field coils and the optical trap are aligned within 50 *μ*m to minimize short-term magnetic field fluctuation during modulation (see supplementary material).

### Dipole oscillations

**Figure 2:**
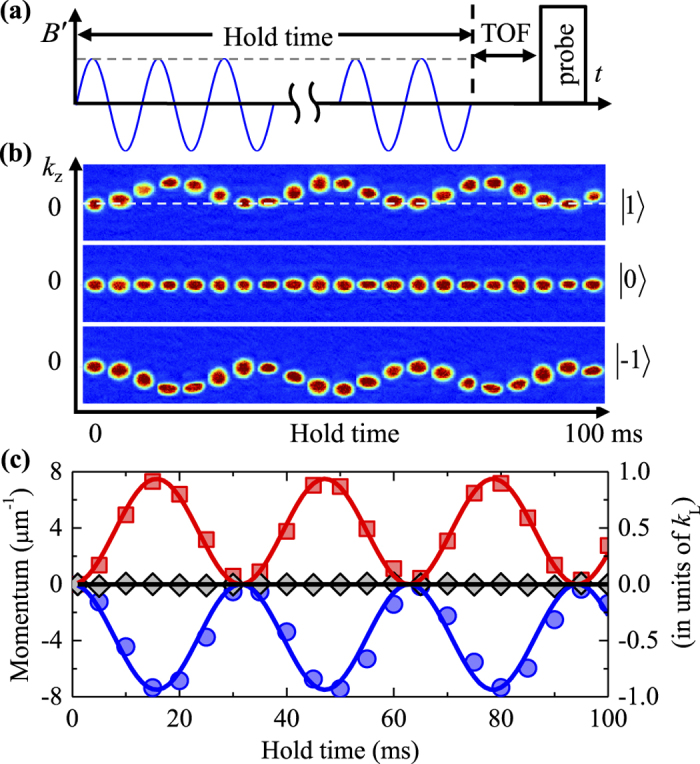
Collective dipole oscillation of a single spin component condensate observed after abruptly turning on *k*_so_ = 7.5 *μm*^−1^ with *ω*_mod_ = (2*π*) 1.0 kHz. (**a**) The time sequence of our experiments: abruptly turning on a constant amplitude modulation GMF corresponding to *k*_so_ = 7.5 *μm*^−1^, followed by 24 ms of time of flight (TOF) before Stern-Gerlach imaging. (**b**) Absorption images of 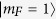 (top row), 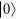 (middle row), and 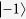 (bottom row) components after different hold time (duration of the modulating GMF). The dashed lines are for *k*_*z*_ = 0, or without SOC. (**c**) Atomic momentum for 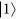 (red squares), 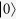 (black rhombuses), and 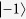 (blue disks) spin components as a function of hold time. The rhs vertical labels are in units of *k*_*L*_, and solid lines denote theoretical predictions.

To confirm the effect of the synthesized SOC from the modulating GMF, we first excite the collective dipole mode of a single spin component atomic condensate in a harmonic trap by abruptly turning on the modulating GMF. By rewriting the effective Hamiltonian (1) as 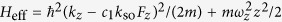 and interchanging the roles of the conjugate variable pair *k*_*z*_ and *z*, it’s easy to find that this effective Hamiltonian is equivalent to that of a particle moving in a displaced harmonic trap. Here, we neglected the extra QZS which only causes an overall energy shift. A particle displaced from the center of a harmonic trap will oscillate back and forth periodically, which is indeed what we observe. Both the position and momentum of the condensate oscillate at the trap frequency *ω*_*z*_. Solving the Heisenberg equations of motion given by *H*_eff_, we obtain the averaged momentum 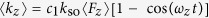, where 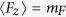 for the 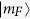 component. The oscillation is around *c*_1_*k*_so_*m*_*F*_ with a peak to peak amplitude 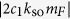. As shown in Fig. 2(a), in our experiments we abruptly turn on the modulating GMF, at a corresponding SOC strength of *k*_so_ = 7.5 *μm*^−1^, and persist for variable hold time. After integer multiple periods of the modulation, the crossed dipole trap is turned off in less than 10 *μ*s. Condensed atoms are expanded for about 24 ms, during which different Zeeman components are Stern-Gerlach separated by an inhomogeneous magnetic field along *x*-direction. For all three spin components, atomic center-of-mass momenta are derived from their shifted positions along *z*-direction with respect to their locations when SOC is absent. As shown in Fig. 2(c), the observed results are in good agreement with our theoretical predictions.

### SOC shifted minimum energy state

**Figure 3:**
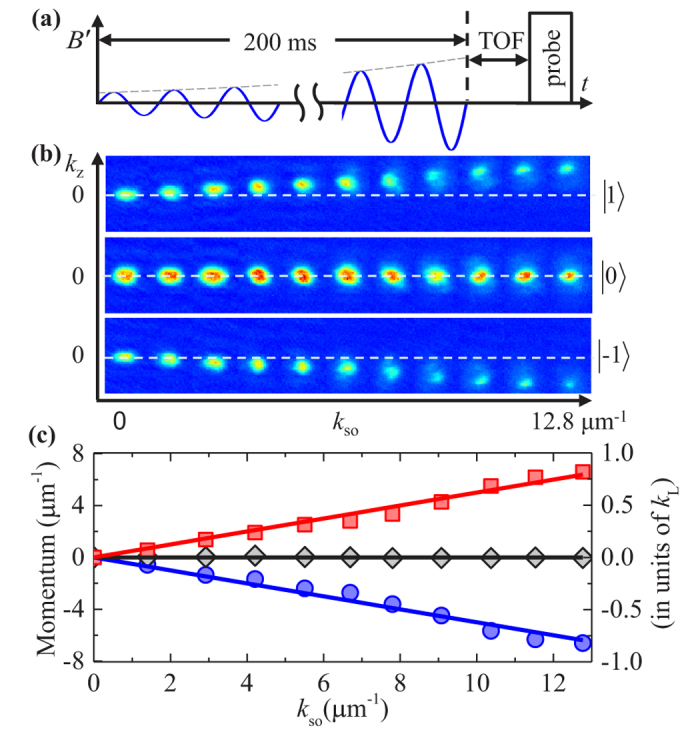
Atoms adiabatically follow the energy minimum shifted to finite momentum with increased SOC strength. Here *ω*_mod_ = (2π) 1.0 kHz. (**a**) The time sequence of our experiments: adiabatically ramping up GMF modulation amplitude to a given *k*_so_, followed by TOF before Stern-Gerlach imaging. (**b**) Absorption images of 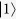 (top row), 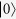 (middle row), 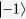 (bottom row) components for different *k*_so_. The dashed lines are for *k*_*z*_ = 0, or for without SOC. With the increase of SOC strength, the observed shapes for the atomic condensate cloud become increasingly distorted, indicating enhanced excitations of higher spatial modes. We believe this is due to the increased violation of the adiabatic condition when ramping the SOC strength to higher strength. Additionally, parametric heating from magnetic field gradient modulation also contributes. (**c**) Atomic center-of-mass momentum for 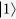 (red squares), 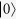 (black rhombuses), and 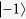 (blue disks) spin component as a function of *k*_so_, compared with theoretical predictions (solid lines).

As a second confirmation, we observe that the atom’s minimum energy state is adiabatically adjusted to a finite none-zero momentum *k*_*z*_ = *c*_1_*k*_so_*m*_*F*_ for the state 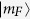 when the modulation amplitude is slowly ramped up as shown in Fig. 3(a). In the presence of SOC, the minimum of the single-particle dispersion for the spin state 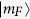 is located at *c*_1_*k*_so_*m*_*F*_. According to the adiabatic theorem, if the ramp of *k*_so_ is slow enough, atoms will follow the ramp and stay at the shifted minimum. In this set of experiments, atoms are prepared at the initial spin state of  
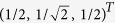
 by applying a *π*/2 pulse to the state (0, 0, 1)^*T*^. The modulation amplitude is then ramped up to a final value within 200 ms. After turning off the optical trap, we measure the momentum for each spin component. We find good agreement with theoretical predictions as shown in Fig. 3(c). To confirm the adiabaticity, *k*_so_ is ramped up from 0 to 4.9 *μm*^−1^ in 100 ms and then back to 0 in another 100 ms. We find atomic center-of-mass momentum returns to 0 without noticeable heating. We also check the dependence of *c*_1_ on the modulation frequency *ω*_mod_ and find that *c*_1_ essentially remains a constant as long as 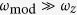.

## Discussion

The results of Fig. 3 demonstrate tunability of the SOC we synthesize. For our protocol based on temporally modulating GMFs, a point of detrimental importance concerns heating which causes relaxation and atom loss from condensates.

As mentioned before, one of the major heating mechanisms for the Raman scheme is photon scattering from the Raman laser. For different alkaline metal atoms, the situation varies significantly. A list of all alkaline metal atom data are shown in Table 1. For cesium and rubidium, the heating rate is very low, the Raman scheme works very well. For potassium, the heating will cause significant problem at the temperature scale of many quantum many body phenomenon. For sodium and lithium, the huge heating rate will quickly destroy the BEC or degenerate Fermi gas.

**Figure 4:**
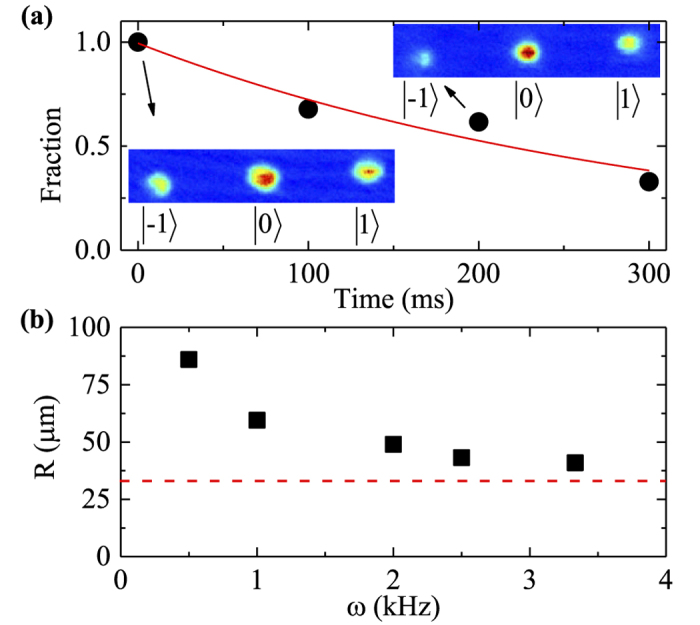
(**a**) The fraction of remaining atoms (due to heating loss from the trap) as a function of the time (after the modulating GMF is turned off) for 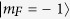 component at *k*_so_ = 4.9 *μm*^−1^. Dots denote measurement points, while the solid line is an exponential fit. The optical trap frequencies are respectively 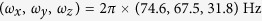, and *ω*_mod_ = (2*π*) 1.0 kHz. The insert Stern-Gerlach images show the three spin components displaced along the vertical direction as a result of spin dependent momentum impulses from SOC; (**b**) The condensate size *R* (fitted radius) after 24 ms TOF expansion (black square) for 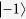 component at *k*_so_ = 4.9 *μm*^−1^ as a function of modulation frequency *ω*_mod_. The dashed line denotes the same size without SOC or at *k*_so_ = 0 *μm*^−1^. Hold time is 100 ms. The optical trap frequencies are respectively 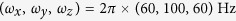.

As atomic spontaneous emission is absent in our experiments, the most likely heating mechanism comes from parametric processes associated with the temporal modulation. To minimize parametric heating, we modulate the GMF far away from the characteristic frequencies of our system. The typical trapping frequency is about (2*π*) 100 Hz and the mean field interaction energy is around (2*π*) 200 Hz. When modulated at *ω*_mod_ = (2*π*) 1.0 kHz, heating is found to be moderate and acceptable for the reported experiments, based on the observed condensate life time. To measure the life time, we adiabatically increase the SOC strength to prepare atoms into an equilibrium state from a initial spin state 
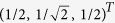
 and measure the fractions of remaining atoms as functions of time. The worst case occurs for condensates in the 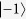 component, whose life time is found to be around 310 ms for *k*_so_ = 4.9 *μm*^−1^ corresponding to a recoil energy of 1.3 kHz, as shown in Fig. 4(a). The life time of our system is comparable to the reported values for the Raman scheme with Rb atoms^11^. It can be further improved by increasing the modulation frequency. Figure 4(b) displays the dependence of the atomic cloud size after 24 ms of TOF expansion on modulation frequency at a fixed *k*_so_ = 4.9 *μm*^−1^. The cloud radius is found to decrease with increasing modulation frequency, which confirms the expected heating suppression with increased modulation frequency. Thus enhanced performances of the GMF scheme is expected if our experiments can be carried out with atomic chip based setups, which routinely provide higher GMFs and faster modulations^58^,^59^.

As demonstrated in our experiments, the SOC synthesized from GMF enacts spin-dependent momentum shifts to the single-particle dispersion curves, leading to curve crossings between different spin states. Inspired by the idea of ref. 50, we find that these crossings can be tuned into avoided crossings when spin flip mechanism is introduced as elaborated in more detail in the supplementary material.

In conclusion, we experimentally demonstrate a tunable SOC synthesized by a modulating GMF for a spin-1 ^87^Rb BEC. We tune the SOC strength by changing the momentum impulse from the GMF. The observed coherence time is reasonably long compared to the Raman scheme for rubidium, and expected to be much better than the Raman scheme for sodium and lithium, pointing to promising experimental opportunities. The scheme we realized relies on spin-dependent Zeeman interactions, thus is naturally extendable to high-spin atomic states, like the spin-1 case we demonstrate here. It adds to the recent report of spin-1 SOC^60^ concatenated from two pseudo-spin-1/2 subsystems with Raman laser couplings. Our experiment opens up a new avenue in the synthesizing SOC with atom species not suitable with the Raman scheme, and presents an important addition to quantum simulation with tunable SOC couple strength.

## Methods

### Condensate production

Atomic ^87^Rb Bose-Einstein condensates are produced employing laser cooling followed by evaporative cooling in a single vacuum chamber, which consists of a 32 mm × 32 mm wide and 300 mm long quartz cell, a Rb reservoir, and vacuum pumps. The quartz cell is ideally suited for magnetic field modulation as it prevents eddy current, which could cause short term fluctuations to the bias field. To optimize the applications of fast and strong GMF modulation by small gradient coils and to allow for three dimensional (3D) optical access, MOT collected from background atoms are magnetically transported 10 cm to a location 8 mm away from the inside wall of the glass cell. They are first RF evaporative cooled in a hybrid trap consisting of a magnetic quadruple trap and a crossed optical dipole trap for 5 seconds. The crossed dipole trap derives from two 1064 nm laser beams (with 1/*e*^2^ Gaussian diameter 120 *μm*) propagating along *x*- and *z*- directions, respectively. The magnetic quadrupole field gradient is then ramped down to transfer atoms to the crossed dipole trap. Condensation occurs after a final evaporation by ramping down the power of the crossed dipole trap in 3 seconds. At the end of the final evaporation the crossed dipole trap frequencies are 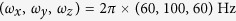. A condensate in 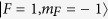 state with 1.2 × 10^5^ atoms is produced every 40 seconds. To minimize heating from the near-resonant driving of the modulating GMF, we further ramp down *ω*_*z*_ to 2*π* × 31.8 Hz in 500 ms after BEC production, which reduces *ω*_*x*_ and *ω*_*y*_ respectively to 2*π* × 74.6 Hz and 2*π* × 67.5 Hz.

### Magnetic field control

Three pairs of Helmholtz coils are used to control the homogeneous bias magnetic field. While transferring atoms from the hybrid trap into the crossed dipole trap, a 0.7 Gauss bias field along *z*-direction is simultaneously turned on in order to maintain atoms in 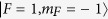 state. In the last 1.5 seconds of evaporative cooling, we ramp up the bias field to 5.7 Gauss and hold on to this value. The Larmor frequency of the bias field is calibrated by RF driven Rabi oscillations between Zeeman sublevels. The residual magnetic field gradient is compensated to below 2 mGauss/cm by a pair of anti-Helmholtz coils along *z*-direction.

A pair of small anti-Helmholtz coils is used to modulate the GMF with a modulation amplitude up to 100 Gauss/cm at a frequency of (2*π*) 1.0 kHz. The radius for the gradient coils is 15 mm. The two coils are separated at an inner distance of 36 mm. Each coil consists of 12 turns of winding and produces an inductance of about 10 *μH*. The gradient coil size is much larger than the 120 *μm* beam waist of our dipole trap, which produces a homogeneous gradient field inside the crossed dipole trap. The coils are small enough to ensure fast and strong GMF modulation. The current for the gradient coils is regulated by a home made fast (10 *μs* rise time) and precise (100 ppm) linear bipolar current controller, whose phase compensation is carefully analysed and tuned for stable running with the inductive load. The gradient coil is mounted on a 3D low magnetic translation stage for precise alignment of the gradient coils. The center of the gradient field is aligned within 50 *μm* with the BEC, which is found to be crucial for minimizing short term bias field fluctuations during GMF modulation.

## Additional Information

**How to cite this article**: Luo, X. *et al.* Tunable atomic spin-orbit coupling synthesized with a modulating gradient magnetic field. *Sci. Rep.* **6**, 18983; doi: 10.1038/srep18983 (2016).

## Supplementary Material

Supplementary Information

## Figures and Tables

**Table 1:** The heating rate for atoms inside a harmonic trap due to spontaneous emission is estimated to be 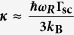, where *ω*_*R*_ is the resonant photon recoil frequency, and *k*_B_ is the Boltzmann constant.

Atomic species	Δ_FS_ (GHz)	Γ (MHz)	*ω*_*R*_ (kHz)	*κ* (nK/s)
^133^Cs	17610	5.2	2.06	0.13
^87^Rb	7123	6.1	3.77	1.2
^40^K	1730	6.0	8.49	25
^23^Na	516	9.8	25	1193
^6^Li	10	5.9	73.9	321167

The effective spontaneous emission rate 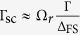 is proportional to the Raman coupling strength Ω_*r*_, where Δ_FS_ is the fine structure splitting in the first excited p state, and Γ is the excited state line width. The heating rate *κ* is calculated for Ω_*r*_ ≈ *ω*_*R*_, which is a typical value used for studying ground state properties of the SOC Hamiltonian^11^.
