# Supplementary material for: Tunable atomic spin-orbit coupling synthesized with a modulating gradient magnetic field
Source: Sci Rep. 2016 Jan 11;6:18983. doi: 10.1038/srep18983 (PMC4707438; doi:10.1038/srep18983)
Supplement: Supplementary Information [file srep18983-s1.pdf]

Supplementary material: Tunable atomic spin-orbit coupling synthesized with a modulating gradient magnetic field

Xinyu Luo,[1] Lingna Wu,[1] Jiyao Chen,[1] Qing Guan,[2] Kuiyi Gao,[2] Zhi-Fang Xu,[3] L. You,[1,4] and Ruquan Wang[2,4,*]

[1]*State Key Laboratory of Low Dimensional Quantum Physics, Department of Physics, Tsinghua University, Beijing 100084, China*

[2]*Beijing National Laboratory for Condensed Matter Physics, Institute of Physics, Chinese Academy of Sciences, Beijing 100080, China.*

[3]*MOE Key Laboratory of Fundamental Physical Quantities Measurements, School of Physics, Huazhong University of Science and Technology, Wuhan 430074, China*

[4]*Collaborative Innovation Center of Quantum Matter, Beijing, China*

PACS numbers: 67.85.De, 03.75.Mn, 67.85.Jk

* ruquanwang@iphy.ac.cn

This supplemental material addresses a few points of the main text in more detail.

THE EFFECTIVE HAMILTONIAN WITH A MODULATING GRADIENT MAGNETIC FIELD

We first discuss how a one dimensional (1D) gradient magnetic field (GMF) is created. The effective Hamiltonian with synthesized spin-orbit coupling (SOC) is then derived for atoms moving in a modulating GMF [1].

As demonstrated in the main text, the key element for synthesizing SOC is the spin rotation transformation $\mathcal{U}_z = \exp\{-ik_{\rm so}zF_z\}$, which can be realized by a 1D GMF and the corresponding rotation angle scales linearly with z-coordinate. According to Maxwell's equations, both the divergence and curl of a static magnetic field vanish in free space. Hence a 1D GMF $B'z\hat{z}$ cannot exist alone. However, as we show below, this can be approximately produced by a combination of a pulsed 3D quadruple field $B'(t)(-x\hat{x}/2 - y\hat{y}/2 + z\hat{z})$ with a constant strong bias field $B_0\hat{z}$. Neglecting quadratic Zeeman shift (QZS), the interaction between atomic spin and magnetic field is described by $H_B^{(0)} = g_F\mu_B\mathbf{F}\cdot\mathbf{B} = g_F\mu_B\mathbf{F}\cdot\mathbf{n}|B|$ where $\mathbf{n}$ is the magnetic field direction. If $B_0 \gg B'(t)y/2, B'(t)x/2$, the magnetic field direction is approximately slaved to the z-axis or $\mathbf{n}\approx\hat{z}$. The magnetic field strength

$$\begin{aligned}|B| &= \sqrt{[B_0 + B'(t)z]^2 + [B'(t)x/2]^2 + [B'(t)y/2]^2}\\ &\approx B_0 + B'(t)z + \frac{B'^2(t)}{8B_0}(x^2+y^2)\\ &\approx B_0 + B'(t)z,\end{aligned} \tag{1}$$

then gains a term linearly proportional to z-coordinate, or approximately $H_B^{(0)} \approx g_F\mu_B[B_0 + B'(t)z]F_z$.

The strong bias field induces spin precession at the Larmor frequency $\omega_0 = g_F\mu_B B_0/\hbar$, which can be transformed away in a frame rotating around z-axis at a frequency $\omega = \omega_0$. The transformed Hamiltonian H_B in the rotating frame corresponds to

$$\begin{aligned}H_B &= R_t H_B^{(0)} R_t^\dagger - i\hbar R_t\partial_t R_t^\dagger\\ &= g_F\mu_B B'(t)zF_z,\end{aligned} \tag{2}$$

where $R_t = \exp(i\omega t F_z)$. Thus we generate an effective 1D GMF $B'(t)z\hat{z}$.

Now consider an atom moving in a periodically modulating 1D GMF $\mathbf{B}(t) = \beta(t)(\hbar k_{\rm so}/g_F\mu_B)z\hat{z}$ where $\beta(t+T) = \beta(t)$, $\int_0^T \beta(t)dt = 0$, and $\hbar k_{\rm so}$ is generalized to the oscillation amplitude of atomic

momentum during modulation. The dynamics for an atom is described by the Hamiltonian

$$H(t) = \frac{\hbar^2 k_z^2}{2m} + \beta(t)(\hbar k_{\mathrm{so}}) z F_z. \tag{3}$$

After a unitary transformation $R_z(t) = \exp\left[i z k_{\mathrm{so}} F_z \int_0^t \beta(t_1)\, dt_1\right]$, the above Hamiltonian is changed into

$$\begin{aligned} \tilde{H}(t) &= R_z(t) H(t) R_z^\dagger(t) - i\hbar R_z(t) \partial_t R_z^\dagger(t) \\ &= \frac{\hbar^2}{2m}\left(k_z - k_{\mathrm{so}} F_z \int_0^t \beta(t_1)\, dt_1\right)^2. \end{aligned} \tag{4}$$

Because $[\tilde{H}(t), \tilde{H}(t')] = 0$, the time evolution operator in the rotated frame over one period takes a simple form $\tilde{U}(T) = \exp[-i\int_0^T \tilde{H}(t)dt/\hbar]$. As $\int_0^T \beta(t)dt = 0$ or $R_z(T)$ becomes the identity operator. Thus after one period of evolution, wavefunctions in the two frames can only differ by a phase factor. The effective Hamiltonian for the whole period reads

$$\begin{aligned} H_{\mathrm{eff}} &= \frac{1}{T}\int_0^T \tilde{H}(t)dt \\ &= \frac{\hbar^2 k_z^2}{2m} - \frac{c_1 \hbar^2 k_{\mathrm{so}}}{m} k_z F_z + \frac{c_2 \hbar^2 k_{\mathrm{so}}^2}{2m} F_z^2, \end{aligned} \tag{5}$$

where $c_n = \int_0^T dt[\int_0^t \beta(t')\mathrm{d}t']^n/T$. For the special case of a sinusoidal GMF $B'(t) = (\pi/T)\sin(2\pi t/T + \varphi)$ with an initial phase φ, one easily arrives at $c_1 = (1/2)\cos\varphi$ and $c_2 = 3/8$. In our experiments $\varphi = 0$ is used, thus $c_1 = 1/2$ and $c_2 = 3/8$. The above derivation neglects QZS from the bias field $\hbar q F_z^2$, because it commutes with Eq. (5), thus can be directly absorbed into the effective Hamiltonian

$$H_{\mathrm{eff}} = \frac{\hbar^2 k_z^2}{2m} - \frac{c_1 \hbar^2 k_{\mathrm{so}}}{m} k_z F_z + (\hbar q + \frac{c_2 \hbar^2 k_{\mathrm{so}}^2}{2m}) F_z^2, \tag{6}$$

THE EFFECTIVE HAMILTONIAN WITH RADIO-FREQUENCY MAGNETIC FIELD PULSES

In our experiment, a modulating GMF generates an effective 1D SOC which does not contain any explicit spin flip interaction. If wanted, spin flip interaction can be introduced from additional RF pulses, following an earlier suggestion in Ref. [2]. Fig. S1(a) illustrates schematically the new scheme. Each period is now composed of two parts with durations T_1 and T_2 respectively for pulsed interactions with GMF and RF magnetic fields.

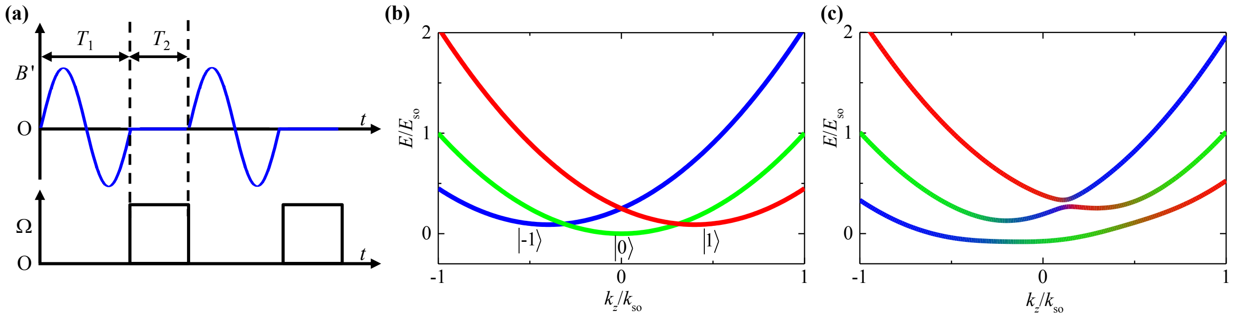

FIG. S1. Spin flip coupling synthesized from interlacing (a) sinusoidal modulations for the GMF (blue wavy lines) of duration T_1 with radio-frequency (RF) couplings (black rectangles) of duration T_2. (b) The dispersion curves of $|1\rangle$ (red solid line), $|0\rangle$ (green solid line), and $|-1\rangle$ (blue solid line) states in the absence of RF coupling for $\tilde{c}_1 = 0.4$, $\hbar\tilde{\Omega} = 0E_{\rm so}$, $\hbar\Delta = 0E_{\rm so}$, $\hbar\tilde{q} = 0.25E_{\rm so}$, in the rotating frame at a frequency $\omega = \omega_0$. (c) The avoided crossing dispersion curves in the presence of RF coupling for $\tilde{c}_1 = 0.4$, $\hbar\tilde{\Omega} = 0.15E_{\rm so}$, $\hbar\Delta = -0.1E_{\rm so}$, $\hbar\tilde{q} = 0.25E_{\rm so}$. The colors for the lines are now given by the weighted averages of the $|1\rangle$, $|0\rangle$, and $|-1\rangle$ components in the eigenstates of Eq. (12) respectively as red, green, and blue colors, this time in the rotating frame at a frequency $\omega = \omega_{\rm RF}$.

The interaction between an atom and the 3D quadrupole magnetic field, the bias field, and the RF magnetic field $b(t)\cos(\omega_{\rm RF}t)\hat{x}$ is governed by the Hamiltonian

$$H_B^{(0)} = g_F\mu_B B'(t)(-xF_x/2 - yF_y/2 + zF_z) + \hbar\omega_0 F_z + g_F\mu_B b(t)\cos\omega_{\rm RF}tF_x + \hbar qF_z^2. \quad (7)$$

In the frame rotating around z-axis at a frequency $\omega = \omega_{\rm RF}$, it is transformed to

$$\begin{aligned} H_B =& R_t H_B^{(0)} R_t^\dagger - i\hbar R_t\partial_t R_t^\dagger \\ =& -g_F\mu_B B'(t)x[\cos(\omega_{\rm RF}t)F_x + \sin(\omega_{\rm RF}t)F_y]/2 - g_F\mu_B B'(t)y[\cos(\omega_{\rm RF}t)F_y - \sin(\omega_{\rm RF}t)F_x]/2 \\ &+[g_F\mu_B B'(t)z + \hbar\omega_0 - \hbar\omega_{\rm RF}]F_z + \frac{1}{2}g_F\mu_B b(t)\{[1+\cos(2\omega_{\rm RF}t)]F_x + \sin(2\omega_{\rm RF}t)F_y\} + \hbar qF_z^2, \quad (8) \end{aligned}$$

where $|q| = \omega_0^2/\Delta_{\rm HFS}$ is the bias field QZS, and $\Delta_{\rm HFS}$ denotes the ground state hyperfine splitting. If $\omega_{\rm RF} \gg g_F\mu_B B'y/(2\hbar)$ and $g_F\mu_B B'x/(2\hbar)$, which is equivalent to $B_0 \gg B'x/2$ and $B'y/2$ as $\omega_{\rm RF} \approx \omega_0$, all fast rotating terms at frequencies $\omega_{\rm RF}$ and $2\omega_{\rm RF}$ can be neglected, thus the Hamiltonian reduces to

$$H_B \approx [g_F\mu_B B'(t)z - \hbar\Delta]F_z + \hbar\Omega(t)F_x + \hbar qF_z^2, \quad (9)$$

where $\Delta = \omega_{\rm RF} - \omega_0$ is the detuning of the RF field frequency from the linear Zeeman shift, $\Omega(t) = g_F\mu_B b(t)/2\hbar$ is the corresponding resonant Rabi frequency of the RF field.

Including the kinetic term, the evolution operator for one period is given by

$$U(T) = U_{\mathrm{RF}}(T_2)U_{\mathrm{so}}(T_1), \tag{10}$$

where $U_{\mathrm{so}}(T_1)$ and $U_{\mathrm{RF}}(T_2)$ are the evolution operators for the two parts respectively. In the first part a periodically modulating GMF is applied with $\Omega = 0$. Following the same steps outlined in the previous section, we obtain $U_{\mathrm{so}}(T_1) = \exp\{-iH_{\mathrm{so}}T_1/\hbar\}$, where

$$H_{\mathrm{so}} = \hbar^2 k_z^2/(2m) - c_1\hbar^2 k_{\mathrm{so}} k_z F_z/m - \hbar\Delta F_z + (\hbar q + c_2 E_{\mathrm{so}})F_z^2, \tag{11}$$

with $E_{\mathrm{so}} = \hbar^2 k_{\mathrm{so}}^2/2m$. In the second part, the quadruple field is turned off with $B' = 0$. According to Eq. (9), we obtain $U_{\mathrm{RF}}(T_2) = \exp\{-iH_{\mathrm{RF}}T_2/\hbar\}$, where

$$H_{\mathrm{RF}} = \hbar^2 k_z^2/(2m) + \hbar\Omega F_x - \hbar\Delta F_z + \hbar q F_z^2. \tag{12}$$

When the pulses are sufficiently short, we can simply add the noncommuting exponents to arrive at $U(T) \approx \exp\{-i\,[H_{\mathrm{so}}T_1/T + H_{\mathrm{RF}}T_2/T]\,T/\hbar\}$. Thus the effective Hamiltonian for a complete period is given by

$$H_{\mathrm{eff}} = \frac{\hbar^2 k_z^2}{2m} - \frac{\tilde{c}_1\hbar^2 k_{\mathrm{so}}}{m} k_z F_z + \hbar\tilde{\Omega} F_x - \hbar\Delta F_z + \hbar\tilde{q} F_z^2, \tag{13}$$

where $\tilde{c}_1 = c_1 T_1/T$, $\hbar\tilde{q} = \hbar q + c_2 E_{\mathrm{so}} T_1/T$, and $\tilde{\Omega} = \Omega T_2/T$. A gap now opens up between different eigenstate dispersions as is shown in Fig. S1(c). This fine tuning is essential to the rich physics already probed in previous SOC experiments [3–9], and constitutes one of the most important future directions for the present experiment.

In this generalized scheme, the SOC term $\propto k_z F_z$ and the spin flip term $\propto F_x$ do not commute with each other, thus the net gauge field in Eq. (13) cannot be transformed away. Our protocol augmented with RF pulses can be understood intuitively. An atom moves a distance depending on its spin state after one period of gradient magnetic field pulses. It acquires a spin dependent group velocity (dz/dt) due to the modulating GMF while its canonical momentum (p_z) remains unchanged. It is this step that couples atomic spin and velocity. The additional RF pulses then couples different spin states. This coupling is also immune to atomic spontaneous emission, like GMF induced coupling. The two steps together give a velocity changing spin coupling, which can be contrasted to the Raman scheme, where SOC is accomplished by a momentum sensitive two-photon Raman transition. Thus our scheme (with additional RF pulses) and the Raman scheme are equivalent in their physical effects.

ESTIMATION OF ENERGY SCALE

The characteristic energy scales for the SOC and the effective QZS are both of the order of atomic recoil energy $E_{\rm so} = \hbar^2 k_{\rm so}^2/2m$, which is typical for most ultracold atomic quantum gas systems. For the particular parameters adopted in our study, this is about 1 kHz for $k_{\rm so} \sim 0.5k_L$ (one photon recoil momentum). The bias field QZS is about 1 kHz. The coherent spin exchanging collision interaction at atomic densities of our experiment is about 10 Hz for ^{87}Rb atoms in $F = 1$ states. The relaxation or dissipation is mainly caused by parametric heating from the time modulating GMF, which we estimate based on the inverse of the observed life time (300 ms for $k_{\rm so} \sim 0.5k_L$), to be about 3Hz, much smaller than the characteristic energy of SOC. Three-body loss is negligible under our experimental conditions. This is affirmed by our observed life time, which is long enough to observe interesting phenomena associated with SOC. In our experiments, the linear Zeeman splitting or the distance between the states $|1\rangle$, $|0\rangle$, $|-1\rangle$ is about 4 MHz, which is much larger than other energy scale, due to the strong bias magnetic field used to select out the one dimensional gradient. The largest energy scale of our system as implemented is the bias field Zeeman energy, which is much larger than the coherent spin coupling, thus prevented us from directly observing spin flip oscillations. In section II we present a generalized scheme to resolve this difficulty by introducing a near resonant coherent drive coupling different atomic spin states, which could nullify the off scale Zeeman shift, as employed in the Raman scheme. Besides, even with a large Zeeman shift, we can still observe interesting many-body phenomena. For example, coherent spin dynamics where two atoms in the $|0\rangle$ state oscillate into a pair of atoms in the $|1\rangle$ and $|-1\rangle$ states was observed experimentally with linear Zeeman shift much larger than coherent spin exchange, due to conservation of the spin magnetization [10].

---

[1] Anderson, B. M., Spielman, I. B., & Juzeliunas, G. Magnetically generated spin-orbit coupling for ultracold atoms. *Phys. Rev. Lett.* **111**, 125301 (2013).

[2] Struck, J., Simonet, J., & Sengstock, K. Spin-orbit coupling in periodically driven optical lattices. *Phys. Rev. A* **90**, 031601 (2014).

[3] Lin, Y.-J., Jiménez-García, K., & Spielman, I. B. Spin-orbit-coupled bose-einstein condensates. *Nature* **471**, 83–86 (2011).

[4] Zhang, J.-Y. *et al.* Collective dipole oscillations of a spin-orbit coupled bose-einstein condensate. *Phys. Rev. Lett.* **109**, 115301 (2012).

[5] Wang, P. *et al.* Spin-orbit coupled degenerate fermi gases. *Phys. Rev. Lett.* **109**, 095301 (2012).

[6] Cheuk, L. W. *et al.* Spin-injection spectroscopy of a spin-orbit coupled fermi gas. *Phys. Rev. Lett.* **109**, 095302 (2012).

[7] Beeler, M. C. *et al.* The spin hall effect in a quantum gas. *Nature* **498**, 201–204 (2013).

[8] Fu, Z. *et al.* Production of feshbach molecules induced by spin-orbit coupling in fermi gases. *Nat. phys.* **10**, 110–115 (2014).

[9] Ji, S.-C. *et al.* Experimental determination of the finitetemperature phase diagram of a spin-orbit coupled bose gas. *Nat. phys.* **10**, 314–320 (2014).

[10] Chang, M.-S., Qin, Q., Zhang, W., You, L., & Chapman, M. S. Coherent spinor dynamics in a spin-1 bose condensate. *Nat. Phys.* **1**, 111–116 (2005).
